# Temporal trends of COVID-19 antibodies in vaccinated healthcare workers undergoing repeated serological sampling: An individual-level analysis within 13 months in the ORCHESTRA cohort

**DOI:** 10.3389/fimmu.2022.1079884

**Published:** 2023-01-11

**Authors:** Giulia Collatuzzo, Giuseppe De Palma, Francesco S. Violante, Stefano Porru, Francesca Larese Filon, Eleonora Fabianova, Concepción Violán, Luigi Vimercati, Mihaela Leustean, Marta Maria Rodriguez-Suarez, Emanuele Sansone, Emma Sala, Carlotta Zunarelli, Vittorio Lodi, Maria Grazia Lourdes Monaco, Gianluca Spiteri, Corrado Negro, Jana Beresova, LucÌa A. Carrasco-Ribelles, Silvio Tafuri, Shuffield S. Asafo, Giorgia Ditano, Mahsa Abedini, Paolo Boffetta

**Affiliations:** ^1^ Department of Medical and Surgical Sciences, University of Bologna, Bologna, Italy; ^2^ Department of Medical and Surgical Specialties, Radiological Sciences and Public Health, University of Brescia, Brescia, Italy; ^3^ Occupational Medicine Unit, IRCCS Azienda Ospedaliero-Universitaria di Bologna, Bologna, Italy; ^4^ Section of Occupational Medicine, Department of Diagnostics and Public Health, University of Verona, Verona, Italy; ^5^ Unit of Occupational Medicine, University of Trieste, Trieste, Italy; ^6^ Occupational Health Department, Regional Authority of Public Health, Banská Bystrica, Slovakia; ^7^ Unitat de Suport a la Recerca Metropolitana Nord, Institut Universitari d’Investigació en Atenció Primària Jordi Gol (IDIAP Jordi Gol), Mataró, Spain; ^8^ Direcció d’Atenció Primària Metropolitana Nord Institut Català de Salut, Barcelona, Spain; ^9^ Germans Trias i Pujol Research Institute (IGTP), Badalona, Spain; ^10^ Universitat Autònoma de Barcelona, Cerdanyola del Vallès, Spain; ^11^ Interdisciplinary Department of Medicine, University of Bari, Bari, Italy; ^12^ National Institute of Public Health, Bucharest, Romania; ^13^ Instituto de Investigación Sanitaria del Principado de Asturias (ISPA) and Universitario Central de Asturias (HUCA), University of Oviedo, Oviedo, Spain; ^14^ Unit of Occupational Health, Hygiene, Toxicology and Prevention, ASST Ospedali Civili di Brescia, Brescia, Italy; ^15^ Occupational Medicine Unit, University Hospital of Verona, Verona, Italy; ^16^ Stony Brook Cancer Center, Stony Brook University, Stony Brook, NY, United States

**Keywords:** serial serology, antibodies, temporal trends, COVID-19 vaccination, health care workers

## Abstract

**Short summary:**

We investigated changes in serologic measurements after COVID-19 vaccination in 19,422 subjects. An individual-level analysis was performed on standardized measurements. Age, infection, vaccine doses, time between doses and serologies, and vaccine type were associated with changes in serologic levels within 13 months.

**Background:**

Persistence of vaccine immunization is key for COVID-19 prevention.

**Methods:**

We investigated the difference between two serologic measurements of anti-COVID-19 S1 antibodies in an individual-level analysis on 19,422 vaccinated healthcare workers (HCW) from Italy, Spain, Romania, and Slovakia, tested within 13 months from first dose. Differences in serologic levels were divided by the standard error of the cohort-specific distribution, obtaining standardized measurements. We fitted multivariate linear regression models to identify predictors of difference between two measurements.

**Results:**

We observed a progressively decreasing difference in serologic levels from <30 days to 210–240 days. Age was associated with an increased difference in serologic levels. There was a greater difference between the two serologic measurements in infected HCW than in HCW who had never been infected; before the first measurement, infected HCW had a relative risk (RR) of 0.81 for one standard deviation in the difference [95% confidence interval (CI) 0.78–0.85]. The RRs for a 30-day increase in time between first dose and first serology, and between the two serologies, were 1.08 (95% CI 1.07–1.10) and 1.04 (95% CI 1.03–1.05), respectively. The first measurement was a strong predictor of subsequent antibody decrease (RR 1.60; 95% CI 1.56–1.64). Compared with Comirnaty, Spikevax (RR 0.83, 95% CI 0.75–0.92) and mixed vaccines (RR 0.61, 95% CI 0.51–0.74) were smaller decrease in serological level (RR 0.46; 95% CI 0.40–0.54).

**Conclusions:**

Age, COVID-19 infection, number of doses, time between first dose and first serology, time between serologies, and type of vaccine were associated with differences between the two serologic measurements within a 13-month period.

## Introduction

Vaccines are of utmost importance for human health and are one of the first medical interventions that everyone receives at birth (as long as they are available in the specific setting).

Whereas vaccinations in childhood are usually long lasting, with Measles–Mumps–Rubella (MMR), polio, and yellow fever vaccines conferring lifelong immunity, vaccination effects in adults are generally short lived (1).

Immune memory depends on the type of vaccine, and vaccine-specific immunity can vary in different subjects depending on individual and environmental factors (2). Live attenuated vaccines are highly effective in providing lifelong protection, whereas glycoconjugate vaccines’ immunity duration derives from the characteristics of their carrier. Remarkably, non-adjuvanted vaccines provide sufficient protection against seasonal influenza because the population is already primed by previous infection and vaccination; conversely, new virus strains to which people are immunologically naïve require adjuvanted vaccines and remain a possible cause of new pandemics (2). As reviewed by Castellino and coworkers, the Highly Pathogenic Avian Influenza 1 (H5N1) influenza strain, which spread in 1997 after its first occurrence in Hong Kong, was effectively contained by MF59 adjuvanted vaccines, which were found to provide high levels of protection against H5N1 6 months after the administration of just one boost (3). Messenger RNA (mRNA) vaccines have been extensively studied in recent decades and have been determined to be an effective recourse in times of extreme need, given their rapid manufacturing process (4). Indeed, mRNA technology—Comirnaty and Spikevax—became the protagonist of the COVID-19 pandemic and was crucial in controlling the infection worldwide. Viral vector vaccines, e.g., Vaxzevria, were also put on the market and represent a novel approach that could be implemented during future pandemics (5).

The introduction of vaccines against COVID-19 was pivotal in effecting a substantial reduction of cases of symptomatic disease and the number of hospitalizations and deaths (5–8). However, the administration of additional boosters with the aim of prolonging their protective effect has proven to be necessary. This may be due to the suboptimal duration of immunization conferred by available vaccines and the appearance of new COVID-19 variants such as Delta and Omicron (9).

ORCHESTRA, a multicenter prospective cohort of healthcare workers (HCW) from different South-Eastern European countries, was assessed with the aim of investigating COVID-19 infections in hospital personnel.

Previous analyses in this cohort described the determinants of 3- and 6-months serology following COVID-19 vaccination (10, 11). The objective of the present study is to delineate the trends of serologic levels in the first 13 months post vaccination, both overall and by month, in a population of approximately 20,000 HCW based on nine European study populations (five from Italy, two from Spain, one multicentric from Romania, and one multicentric from Slovakia) included in ORCHESTRA.

## Methods

ORCHESTRA is a prospective multicenter cohort of HCW from several European countries [https://orchestra-cohort.eu (12)]. This analysis includes HCW in nine cohorts from four countries; of these, Slovakia and Romania were multicenter cohorts. In Slovakia, participants were HCW and workers from social care facilities in COVID-19 departments from four regions (Banská Bystrica, Bratislava, Ružomberok, and Martin). In Romania, participants were HCW employed at the National Institute of Public Health and worked in four different locations (Bucharest, Iasi, Cluj, and Timisoara). In both cohorts, local teams followed a unique protocol for blood sampling and preparation and shipment of samples to the central laboratory.

Data on sociodemographic characteristics, PCR testing, and vaccination status (including date of vaccination, number of doses, and type of vaccination administered) were either abstracted from medical surveillance records or collected through questionnaires. The levels of anti-COVID-19 S1 antibodies were derived from medical records or generated through *ad hoc* testing. Because the cohorts are included in the European Commission-sponsored Orchestra project, their data have undergone extensive harmonization. Methods of measurement of the antibody levels varied between the included centers and in the time periods. Different analytical methods were used for different cohorts. We therefore log transformed the results and then we divided them by the standard errors (SE) of each cohort-specific mean. We used the same approach in previous analyses within ORCHESTRA (10, 11).

Selected characteristics of the study population are described in **Table 1**. This study comprises 19,923 HCW from Italy (Bari, Bologna, Brescia, Trieste, and Verona), Spain (Northern Metropolitan Area of Barcelona (Barcelona) and Oviedo), Romania (multicentric), and Slovakia (multicentric), with multiple serologies during a 13-month time frame from first dose administration (between December 2020 and March 2021, depending on the cohort), defined as the interval 150–210 days, including the 13-months serology. After excluding 501 HCW who received fewer than two vaccine doses, 19,422 subjects were included in the analysis.

**Table 1:** Distribution of healthcare workers (HCW) by selected characteristics and mean difference between first and second serologic measurements.

Variable	N (%)	Mean difference* (SE)
**Cohort**		
Italy, Bari	157 (0.8)	1.21 (0.07)
Italy, Bologna	5,536 (28.6)	–0.28 (0.01)
Italy, Brescia	5,881 (30.4)	–0.33 (0.00)
Italy, Trieste	2,058 (10.6)	–0.69 (0.04)
Italy, Verona	4,569 (23.6)	–0.19 (0.01)
Romania, multicenter	67 (0.3)	–0.85 (0.08)
Slovakia, multicenter	583 (3.0)	–0.84 (0.05)
Spain, Barcelona	480 (2.5)	0.37 (0.08)
Spain, Oviedo	33 (0.2)	–0.78 (0.13)
**Sex**		
Male	5,295 (27.3)	–0.29 (0.01)
Female	14,069 (72.7)	–0.31 (0.01)
**Age**		
≤ 29	2,263 (11.7)	–0.36 (0.01)
30–39	4,288 (22.1)	–0.36 (0.01)
40–49	4,714 (24.3)	–0.33 (0.01)
≥ 50	8,099 (41.8)	–0.23 (0.01)
**Job title**		
Administration	1,568 (8.1)	–0.25 (0.03)
Physician (including residents)	4,989 (25.8)	–0.27 (0.01)
Nurse	7,310 (37.7)	–0.33 (0.01)
Technician	1,622 (8.4)	–0.25 (0.03)
Other HCW (including auxiliary workers)	3,875 (20.0)	–0.34 (0.02)
**Previous COVID-19 infection**		
Never infected	16,634 (85.9)	–0.29 (0.01)
Infected before first serologic measurement	2,327 (12.0)	–0.62 (0.02)
Infected between first and second serologic measurement	376 (1.9)	1.01 (0.07)
Infected at both times	27 (0.1)	0.96 (0.29)
**Number of vaccine doses**		
2 doses	18,196 (94.0)	–0.38 (0.01)
3 doses	1,168 (6.0)	0.92 (0.04)
**Time between first and second serologic measurements**		
< 30d	13 (0.0)	0.74 (0.77)
30d–60d	173 (0.9)	–0.37 (0.06)
60d–90d	649 (3.3)	–0.34 (0.03)
90d–120d	3,162 (16.3)	–0.30 (0.01)
120d–150d	5,548 (28.6)	–0.36 (0.01)
150d–180d	1,806 (9.3)	–0.48 (0.02)
180d–210d	4,823 (24.9)	–0.44 (0.02)
210d–240d	1,780 (9.2)	–0.30 (0.03)
240d–270d	603 (3.1)	0.23 (0.06)
270d–300d	426 (2.2)	0.95 (0.07)
300d–330d	274 (1.4)	1.01 (0.08)
330d–360d	71 (0.4)	0.68 (0.15)
360d–390d	19 (0.1)	1.25 (0.21)
390d–410d	16 (0.1)	1.92 (0.38)
410d–440d	1 (0.0)	0.89 (–)
**Time between first serologic measurement and first dose of vaccine**		
<90d	9,989 (51.6)	–0.29 (0.01)
≥90d	9,375 (48.4)	–0.32 (0.01)

* Difference in cohort-specific standardized antibody level.

SE, standard error.

The outcome of this analysis was the difference between the last and first serologic measurement of antibodies in the period 1–13 months from administration of the first dose of the vaccine. To avoid combining both positive and negative differences, we subtracted the observed difference from that of the subject with the highest increase between the two measurements. The analyses aimed at examining this difference according to HCW and vaccine characteristics, including age, sex, study center, previous COVID-19 infection, number of doses, and type of vaccine.

We first conducted descriptive analyses of the outcome and explanatory variables. The main analysis involved calculating the difference in serologic response within 13 months. Multiple linear regression models were used to calculate the relative risks (RR) and their 95% confidence intervals (CI) for the difference between the last and first serologic measurement. Results are expressed as RR for one logarithmic unit in the difference of one standard error of the cohort-specific distribution between the last and the first serologic measurement. Therefore, RRs greater than one correspond to a larger decrease in serologic level between the last and the first measurement compared with the reference category, and vice versa. We performed a secondary analysis that was restricted to HCW who received only two vaccine doses, because most subjects with an increase in antibody levels between the last and first measurements had received three doses of the vaccine, with the last measurement taken after the third dose.

The models included terms for cohort, sex, age (10-year increase), job title, number of vaccine doses received, previous COVID-19 infection, time difference between first serology measure and first dose of vaccine (30-day increase), and time difference between first serology measure and second serology measure (30-day increase).

Stata^®^ software 16 (StataCorp LP, College Station, Texas, USA) was used for the statistical analysis.

The study was approved by the Italian Medicine Agency (AIFA) and the Ethics Committee of the Italian National Institute of Infectious Diseases (INMI) Lazzaro Spallanzani.

## Results

Overall, our analysis included 19,422 HCW, with repeated measurements performed within 13 months after first dose and with two or more vaccine doses. The distribution of measurements by time since first dose and cohort is shown in **Supplementary Figure 1**. Most of the study population consisted of women (72.6%), and a large proportion of subjects were ≥ 50 years old (42.5%). HCW were mostly nurses (37.7%) and physicians (25.7%). Overall, 85.8% had had no previous COVID-19 infection; the vast majority (94.0%) had received two doses of COVID-19 vaccine, and 1,178 (6.0%) from the Bari, Bologna, Romania, Slovakia, Barcelona, and Oviedo cohorts received a third dose. The time between two consecutive serologic measurements ranged from less than 30 days to 440 days, with 16.3% of measurements being performed between 90 and 120 days, 28.4% between 120 and 150 days, 9.4% between 150 and 180 days, 24.9% between 180 and 210 days, and 9.3% between 210 and 240 days. We stratified the measurements based on whether they were collected more or less than 90 days from the first dose of the COVID-19 vaccine (52.0% vs. 48.0%). Most subjects who received two doses (98.2%) were given Comirnaty; the remaining subjects were given Spikevax and other or mixed vaccines (not shown in detail).

**Figure 1:**
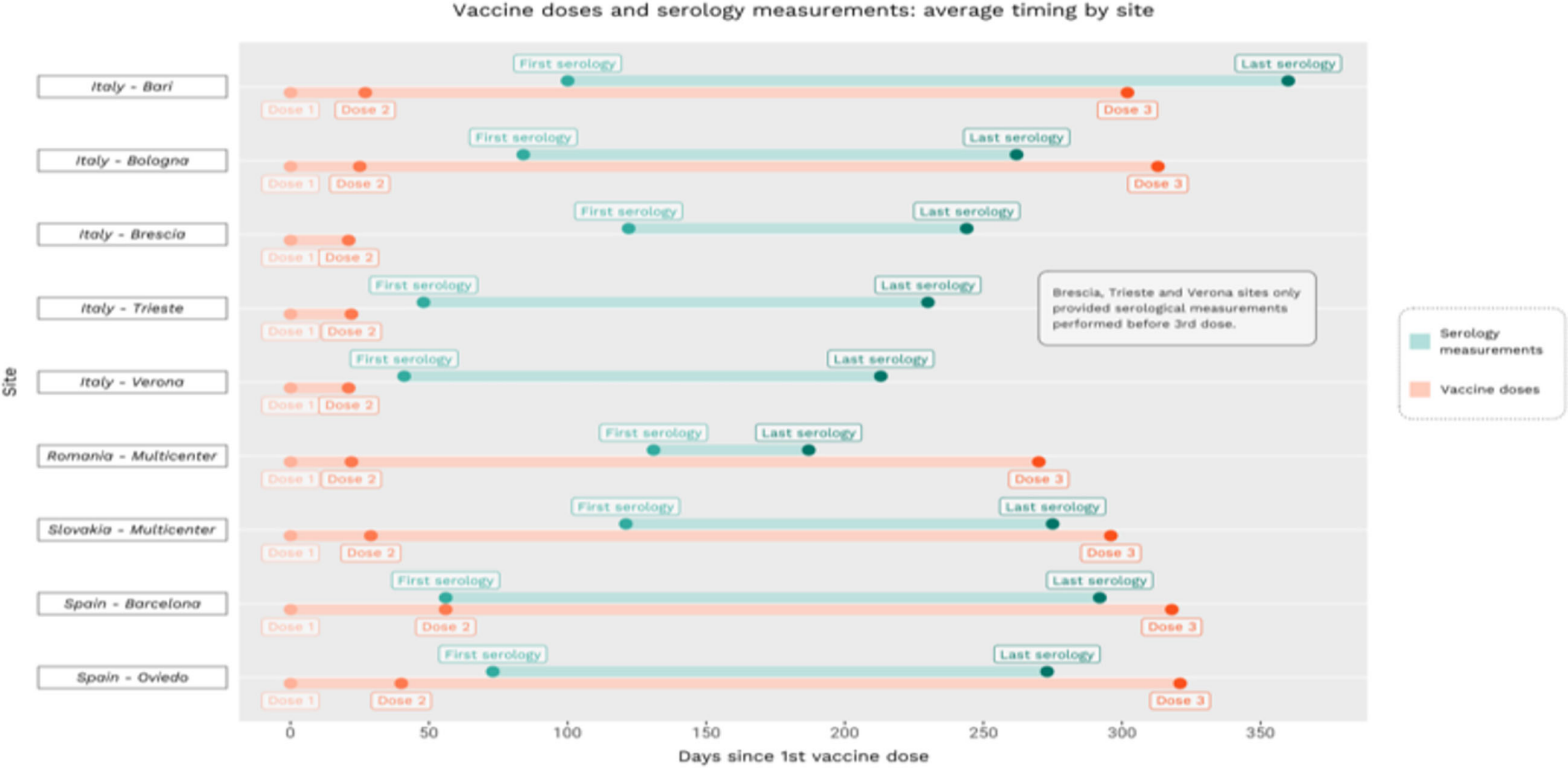
Timing of vaccination and serologic measurements by cohort.

When calculating the mean serology difference between two measurements, we observed some difference by study center, with Bari and Barcelona showing positive differences. This is mainly explained by the fact that several blood samples in these cohorts were collected after the third vaccine dose for a second time. **Figure 1** illustrates the timing of serologic measurements by study center. As the dots contain the average number of days between first and second serology, last serology appears to be infrequently performed after the booster vaccine dose, and blood samples were collected at quite different time points in each center. On the other hand, vaccinations were administered mostly with the same timing, with approximately 30 days between the first and second doses, and the booster dose administered approximately 300 days after first vaccination.

According to the time between the two serologic samples, we calculated a progressively reduced difference from <30 days to 210–240 days (SE 0.74 to –0.30), and a subsequent increase in the difference until days 410–440 (SE 0.22 to 3.10). No substantial difference was observed in the time between first serology and first vaccination (SE –0.30 and –0.32, respectively).

**Table 2** shows the results of the main analysis. Compared with the cohort from Bologna, the cohorts from Bari, Trieste, Verona, Romania, Slovakia, and Barcelona and Oviedo experienced a larger decrease in their antibody levels, and no cohorts experienced a smaller decrease. Age was associated with a larger decrease in serologic levels. No difference was found by sex or job title, and HCW with a history of COVID-19 infection experienced a smaller decrease in antibody levels. The RR for a 30-day increase in time between first vaccine dose and first serology was 1.08 (95% CI 1.07–1.10) and for a 30-day increase between the two samples was 1.04 (95% CI 1.03–1.05). The antibody level at first measurement was a strong predictor of subsequent decrease in the serologic level (RR 1.60, 95% CI 1.56–1.64). Compared with Comirnaty, Spikevax was associated with a smaller decrease in the serologic level (RR 0.83, 95% CI 0.75–0.92), as was the administration of mixed vaccines (RR 0.61, 95% 0.51–0.74). Finally, administration of a third vaccine dose was associated with a smaller decrease in the serologic level (RR 0.46, 95% CI 0.40–0.54).

**Table 2:** Multivariate analysis of characteristics associated with changes in antibody levels between first and second serologic measurement.

Characteristics	RR	95% CI
**Cohort**		
Italy, Bari	1.90	1.53–2.36
Italy, Bologna	Ref	
Italy, Brescia	1.03	0.99–1.06
Italy, Trieste	2.49	2.34–2.64
Italy, Verona	1.68	1.59–1.78
Romania, multicenter	1.80	1.56–2.07
Slovakia, multicenter	2.77	2.55–3.00
Spain, Barcelona	1.81	1.62–2.02
Spain, Oviedo	0.89	0.59–1.33
**Sex**		
Male	Ref	
Female	0.98	0.96–1.00
**Age**		
10 years increase	1.03	1.02–1.04
**Job title**		
Administration	Ref	
Physician (including residents)	1.01	0.97–1.05
Nurse	1.01	0.98–1.05
Technician	0.99	0.95–1.04
Other HCW (including auxiliary workers)	1.01	0.97–1.05
**Previous COVID-19 infection**		
Never infected	Ref	
Infected before the first serologic measurement	0.81	0.78–0.85
Infected between the two serologic measurements	0.33	0.29–0.38
Infected at both times	0.70	0.50–0.99
**Number of vaccine doses**		
2 doses	Ref	
3 doses	0.46	0.40–0.54
**Time between first vaccine dose and first serology measurement**		
30 days increase	1.08	1.07–1.10
**Time between first and second serology measurements**		
30 days increase	1.04	1.03–1.05
**Antibody level at first serologic measurement**		
1 SD increase in ln(antibody level)	1.60	1.56–1.64
**Type of vaccine**		–
Comirnaty	Ref	
Spikevax	0.83	0.75–0.92
Vaxzevria	0.98	0.64–1.50
Mixed vaccines	0.61	0.51–0.74
Missing	0.44	0.37–0.52

RR, relative risk for decrease of one standard error of the cohort-specific distribution of ln(antibody level) between the last and the first serologic measurements, adjusted for all the variables in the table.

CI, confidence interval; HCW, healthcare worker; Ref, reference category; SD, standard deviation.

When the analysis was restricted to HCW who were administered two vaccine doses (N=18,347, **Supplementary Table 1**), the results were comparable to those reported for the whole study population. The only different result was observed for gender, with women exhibiting a smaller decrease in antibody levels than men (RR 0.97, 95% CI 0.95–0.99).

## Discussion

The present study analyzed several potential clinical, individual, and work-related determinants of the difference in serologic level between multiple measurements after COVID-19 vaccination over 13 months in a large European cohort of HCW. Although the effect of sex appeared to be modest, increasing age was associated with a larger decrease in antibody levels. There were no differences according to job title. Previous COVID-19 infection, having received Spikevax or a mixed vaccine, and administration of a third dose of the vaccine resulted in a smaller decrease in antibody levels, whereas an increase in time between first vaccine dose and first serology, and that between the two samples, was associated with the opposite; i.e. a larger decrease in antibody levels. While several of these factors were correlated with one another, the large sample size allowed us to conduct multivariate analyses, whose stable results should exclude reciprocal confounding.

When considering previous COVID-19 infection, a significantly smaller decrease was found in subjects reporting previous infection compared with those never infected. In particular, a one standard error decrease in serologic level was 67% less likely to occur in HCW who became infected between the first and second serologic measurements compared with those who were never infected. This can be explained by the renewal of the immunological peak driven by the infection, leading to a stable antibody level—steadily higher than average—while a difference can be appreciated in naïve subjects, who experience only a decline in the antibody level with time. Among subjects infected before the first serologic sampling. There was a larger difference in serologic level among subjects infected before the first serological sample than among those infected in other time frames, which may be explained by the fact that infection confers a high degree of immunization, thus registering a higher antibody level at the first measurement compared with naïve subjects. Although the effectiveness of COVID-19 vaccines has been debated and given the prevalence of breakthrough infection, when considering our data, the number of infections occurring in vaccinated HCW is approximately one-sixth of the number of infections that occurred before vaccination, corroborating the fact that vaccination is the most important instrument in the prevention of communicable diseases (13–15).

Subjects administered with three doses of the vaccine experienced a 54% lower probability of undergoing a one standard error decrease in antibody level than subjects who had received two doses. This may be due to the timing of the blood sampling, specifically, the overlap between the second measurement and the number of days following the booster dose, which determines the immunological response. Indeed, (i) most of the HCW from Bari underwent their last serology test after their third dose of the vaccine and (ii) on average, in Barcelona, HCW underwent their first serology test at the same time as their second dose of the vaccine (**Figure 1**). This is also captured by the variable related to time between the serologic measurements: between 1 and 7 months the difference in serologic level increases, which implies a rapid decline in the number of antibodies; an increase in serologic levels can be detected between 8 and 13 months. This largely corresponds to the period when HCW were recommended to obtain the third dose of vaccine.

Age was related to the progressive decline in trends of serologic levels, expressed through a 3% higher likelihood of undergoing a one standard error decrease in antibody level with every10-year increase in age. Several studies have demonstrated the lower levels of immunological responsiveness of older subjects (16). This is consistent with our previous study that showed age as a determinant of lower level of serologic response in HCW (11). When focusing on gender differences, a 2% smaller probability of undergoing a one standard error decrease in antibody level was suggested for women, consistent with previous literature (17–19). Indeed, sexual dimorphism in immune responses has been described, which is more evident following infection or vaccination (20, 21).

With regard to job title, no difference was detected. This is likely to be due to the adjustments for different potential confounders which may mediate an effect on occupational categories, because of the different risk exposure among professional healthcare figures. Previous studies focused on HCW as a job category rather than distinguishing potential association with COVID-19 infection and vaccination outcomes. For example, in a previous analysis we once again found no relationship between job title and immunological response (11). Moreover, a previous study based on the Italian cohort of the ORCHESTRA project found no difference in the risk of HCW contracting COVID-19, even when considering HCW in departments dedicated to COVID-19 (22).

The serologic trend was likely to decline with every 30 day increase in time between first vaccination and first serologic measurement, corroborating the evidence of a progressive decline in the level of antibodies and providing a precise quantification of the effect (9). When considering 30 day-time increases between the two serologic measurements, the declining trend was smaller. Moreover, the higher the serologic level detected at the first measurement, the larger the difference detected at the second blood sample.

We found a small difference in the serologic samplings by type of vaccine, namely Comirnaty or Spikevax, with the former leading to a slightly smaller reduction in the antibody level than the latter. However, Spikevax has been shown to confer greater protection in the long term than Comirnaty (23). Based on our analysis, Spikevax resulted in a 20% increase of the serologic level compared with Comirnaty, which is consistent with previous evidence (23). This difference was attributed to a higher mRNA content in Spikevax that in Comirnaty and the longer interval between priming and boosting for Spikevax (4 weeks for Spikevax vs. 3 weeks for Comirnaty) (23). Overall, large differences can be seen among viral vector vaccines and mRNA vaccines (24, 25), and our data did not allow us to address this question in detail.

The description of these trends, together with the evidence of increased difference in antibody level in HCW who received a third vaccine dose, corroborates previous findings on the ability of vaccines to stimulate immunological response (26). Assuming a proportional capacity of the immune system to protect from infection, this implies a greater ability of the vaccines to guarantee protection against COVID-19 infection when booster doses are administered; thus the greater effectiveness of a three-dose scheme of vaccination (27). Despite this, the present analysis can only allow assumptions on the antibody levels as a sign of effective vaccination.

Describing the trends of antibody levels after COVID-19 vaccination is currently a major issue. Indeed, it enables to read the pandemic and the public health interventions which have been introduced to control it in the light of quantitative data, and to understand if and to what extent vaccination schemes have been useful and effective. Vaccines are usually administered in multiple doses (28). Viral pathogens that have a short life cycle and a high rate of replication are subject to higher mutagenicity, leading to continuous exposure to potential infectious risk for both unvaccinated and vaccinated people. This is what commonly happens with influenza viruses, requiring vaccination every year (29). Indeed, HCW in most countries are strongly recommended to receive an influenza vaccination every year as they are occupationally at a higher risk of infection; both for their own protection and to prevent transmission in the hospital environment (30, 31).

The introduction of COVID-19 vaccines into usual medical practice has been hypothesized based on the circumstances registered worldwide (32, 33). The main issue relates to the capacities of the COVID-19 vaccines to reduce the risk of symptomatic disease, and less so to reduce the risk of becoming infected (34).

This study has some strengths and limitations. To our knowledge, this is one of the largest analyses on changes in individual-level data regarding antibody level after COVID-19 vaccination. Most previous studies were based on comparisons of groups of individuals tested at different times after vaccination, including a large prospective study conducted in England on more than 212,000 vaccinated subjects (35). Individual-level serologies were collected in other studies, which, however, included no more than a few hundred participants (36–39). The large sample size, together with the collection of detailed information, allowed assessment of multiple factors associated with trends in antibody levels within 13 months from vaccination, enabling reciprocal adjustment. Moreover, the prospective design of this study enables further follow-up with the participants, offering the possibility to expand this investigation and add interesting insight in future analyses. In addition, this is one of the few studies covering a 13-month period when considering COVID-19 vaccination-related immunity.

The results we obtained are consistent with previous literature (34), showing robustness, and are important in providing further confirmation of individual-level data on the progressive waning of COVID-19 vaccine immunization by enhancing the confidence of these observations. This gives additional strengths to previous data. Furthermore, we added useful information on time-trend serology from first vaccination and in between serologic measurements, as well as comparisons between naïve HCW and those previously infected with COVID-19. We used a strong methodological approach to standardize the level of antibodies, overcoming the issue of different types of tests adopted in the various cohorts, making the measurement from different populations comparable.

The main limitation of this study was the heterogeneity of testing methods used within the cohorts. However, as mentioned above, we addressed this issue by using the standardized log value and calculating RR per one SD increase, thus generating harmonized results on measurements of antibody levels. The same method was used in our previous analysis (11). This approach is particularly valuable given the global connotation of COVID-19 infection, because it allows us to overcome potential heterogeneity in data collection methods and to compare data from different populations. Indeed, all the differences found in the present analysis must be interpreted as representing real phenomena rather than being attributed to differences in serologic testing across the cohorts.

Besides this, our analysis was limited by the small number of HCW who had three doses of the vaccine, leading to a low power of analysis of the effect of the third dose. Moreover, only a small number of HCW had three or more measurements of antibody levels. Thus, we used only the first and the last measurements because most subjects had received only two vaccine doses. Future analysis of this population will include more subjects with three or more measurements of antibody levels, allowing for the assessment of non-linear trends in antibody levels.

We could not distinguish individuals by health status, and therefore could not analyze potential variability in antibody levels due to conditions such as immunodepression. However, this is a working population, representing an overall healthy group, and HCW in particular have been reported to be healthier than the general population (40). In addition, given the paucity of data on different types of vaccine, the possible comparisons were limited in terms of viral vector vaccines.

Furthermore, subclinical infections were not systematically assessed (anti-nucleocapsid antibodies were available for only a subset of participants). This may have diluted the estimated effect of previous COVID-19 infection.

## Conclusions

This analysis defines the trends of serologic levels in HCW who received multiple COVID-19 vaccines within the first 13 months, by calculating the mean difference observed in nine European cohorts. Positive trends were detected in those centers where serologic samples were collected around the time of vaccination, as well as among previously infected HCW. Increasing age, time between first vaccine and first serology, and time between serologies determined a negative trend in antibody level. Spikevax was associated with a smaller decrease in the serologic level than Comirnaty. The booster dose determines the renewal of the immunological response, expressed as a smaller decrease in the serologic level.

These individual-level data support published studies showing a progressive decline of immune response after COVID-19 vaccination. Analyses over longer time frames would be of interest to better understand the longevity of COVID-19 vaccine immunization. Such studies, combined with our cohort, would provide further information on the optimal time frame of administration of subsequent doses. This information should be combined with a risk profile for COVID-19 infection to improve the quantitative information needed to optimize the schedule of vaccine administration, and the subgroups of population to be prioritized for boosters.

Further studies are warranted to further describe temporal changes in serologic levels after COVID-19 vaccination and to clarify the role of different types of vaccines and the timing of infection. To better address the pandemic and manage vaccination strategy at both the occupational and population-based level, studies focused on the protectiveness of vaccination-driven antibodies are needed.

## Data availability statement

The raw data supporting the conclusions of this article will be made available by the authors, without undue reservation upon reasonable request.

## Ethics statement

The study was approved by the Italian Medicine Agency (AIFA) and the Ethics Committee of the Italian National Institute of Infectious Diseases (INMI) Lazzaro Spallanzani. The patients/participants provided written informed consent to participate in this study as well as by local ethics committees.

## Author contributions

GC and PB conceived and designed the study. PB coordinated the international collaboration. PB, FV, VL, GC, and CZ coordinated and conducted the study in Bologna, Italy. GDP, ESan, and ESal coordinated and conducted the study in Brescia, Italy. SP, MM, and GS coordinated and conducted the study in Verona, Italy. FLF and CN coordinated and conducted the study in Trieste, Italy. CV and LC-R coordinated and conducted the study in Barcelona, Spain. ML coordinated and conducted the study in Bucarest, Romania. EF and JB coordinated the study in Slovakia. MR-S coordinated and conducted the study in Oviedo, Spain. LV and ST coordinated and conducted the study in Bari, Italy. MA, GP, and SA performed the statistical analysis. GC and PB supervised the statistical analysis. GC and PB drafted the manuscript. All authors reviewed and approved the final version of the manuscript.
