# Corrigendum: Temporal trends of COVID-19 antibodies in vaccinated healthcare workers undergoing repeated serological sampling: an individual-level analysis within 13 months in the ORCHESTRA cohort

**DOI:** 10.3389/fimmu.2023.1197923

**Published:** 2023-04-14

**Authors:** Giulia Collatuzzo, Giuseppe De Palma, Francesco S. Violante, Stefano Porru, Francesca Larese Filon, Eleonora Fabianova, Concepción Violán, Luigi Vimercati, Mihaela Leustean, Marta Maria Rodriguez-Suarez, Emanuele Sansone, Emma Sala, Carlotta Zunarelli, Vittorio Lodi, Maria Grazia Lourdes Monaco, Gianluca Spiteri, Corrado Negro, Jana Beresova, LucÌa A. Carrasco-Ribelles, Silvio Tafuri, Shuffield S. Asafo, Giorgia Ditano, Mahsa Abedini, Paolo Boffetta

**Affiliations:** ^1^ Department of Medical and Surgical Sciences, University of Bologna, Bologna, Italy; ^2^ Department of Medical and Surgical Specialties, Radiological Sciences and Public Health, University of Brescia, Brescia, Italy; ^3^ Occupational Medicine Unit, IRCCS Azienda Ospedaliero-Universitaria di Bologna, Bologna, Italy; ^4^ Section of Occupational Medicine, Department of Diagnostics and Public Health, University of Verona, Verona, Italy; ^5^ Unit of Occupational Medicine, University of Trieste, Trieste, Italy; ^6^ Occupational Health Department, Regional Authority of Public Health, Banská Bystrica, Slovakia; ^7^ Unitat de Suport a la Recerca Metropolitana Nord, Institut Universitari d’Investigació en Atenció Primària Jordi Gol (IDIAP Jordi Gol), Mataró, Spain; ^8^ Direcció d’Atenció Primària Metropolitana Nord Institut Català de Salut, Barcelona, Spain; ^9^ Germans Trias i Pujol Research Institute (IGTP), Badalona, Spain; ^10^ Universitat Autònoma de Barcelona, Cerdanyola del Vallès, Spain; ^11^ Interdisciplinary Department of Medicine, University of Bari, Bari, Italy; ^12^ National Institute of Public Health, Bucharest, Romania; ^13^ Instituto de Investigación Sanitaria del Principado de Asturias (ISPA) and Universitario Central de Asturias (HUCA), University of Oviedo, Oviedo, Spain; ^14^ Unit of Occupational Health, Hygiene, Toxicology and Prevention, ASST Ospedali Civili di Brescia, Brescia, Italy; ^15^ Occupational Medicine Unit, University Hospital of Verona, Verona, Italy; ^16^ Stony Brook Cancer Center, Stony Brook University, Stony Brook, NY, United States

**Keywords:** serial serology, antibodies, temporal trends, COVID-19 vaccination, health care workers

In the published article, there was an error in the affiliation reference assigned to the author Emanuele Sansone, which name was followed by the number 3 in apex. Instead of “3”, it should be “2”. The correct reference for the author affiliation is:

^2^ Department of Medical and Surgical Specialties, Radiological Sciences and Public Health, University of Brescia, Brescia, Italy.

The authors apologize for this error and state that this does not change the scientific conclusions of the article in any way. The original article has been updated.

