# Supplementary material for: Temporal trends of COVID-19 antibodies in vaccinated healthcare workers undergoing repeated serological sampling: An individual-level analysis within 13 months in the ORCHESTRA cohort
Source: Front Immunol. 2023 Jan 11;13:1079884. doi: 10.3389/fimmu.2022.1079884 (PMC9875291; doi:10.3389/fimmu.2022.1079884)
Supplement: Supplementary file 1 [file Table_1.docx]

Supplementary Table 1. Multivariate analysis of characteristics associated with change in antibody level between last and first serological measurement. Analysis restricted to subjects with two vaccine doses.

| Characteristics | RR | 95% CI |
| --- | --- | --- |
| Cohort |  |  |
| Italy-Bari | 1.35 | 0.98-1.85 |
| Italy-Bologna | Ref |  |
| Italy-Brescia | 1.00 | 0.96-1.03 |
| Italy-Trieste | 2.41 | 2.26-2.56 |
| Italy-Verona | 1.66 | 1.57-1.75 |
| Romania-Multicenter | 1.80 | 1.56-2.06 |
| Slovakia-Multicenter | 2.54 | 2.34-2.75 |
| Spain-Barcelona | 1.82 | 1.63-2.04 |
| Spain-Oviedo | 0.91 | 0.60-1.38 |
| Sex |  |  |
| Male | Ref |  |
| Female | 0.97 | 0.95-0.99 |
| Age |  |  |
| 10 years increase | 1.04 | 1.03-1.05 |
| Job title |  |  |
| Administration | Ref |  |
| Physician (including residents) | 1.00 | 0.96-1.04 |
| Nurse | 1.01 | 0.97-1.05 |
| Technician | 0.99 | 0.94-1.03 |
| Other HCW (including auxiliary workers) | 1.01 | 0.97-1.05 |
| Previous Covid-19 infection |  |  |
| Never infected | Ref |  |
| Infected before the first serological measurement | 0.82 | 0.78-0.86 |
| Infected between the two serological measurements | 0.28 | 0.24-0.33 |
| Infected at both times | 0.61 | 0.35-1.05 |
| Time between first vaccine dose & first serology measurement |  |  |
| 30 days increase | 1.10 | 1.08-1.12 |
| Time between first and second serology measurements |  |  |
| 30 days increase | 1.05 | 1.04-1.06 |
| Antibody level at first serological measurement |  |  |
| 1 SD increase in ln(antibody level) | 1.56 | 1.53-1.60 |
| Type of vaccine |  | - |
| Comirnaty | Ref |  |
| Spikevax | 0.83 | 0.75-0.93 |
| Vaxzervria | 0.99 | 0.64-1.54 |
| Mixed vaccines | 0.55 | 0.40-0.75 |
| Missing | 0.97 | 0.75-1.24 |

RR, relative risk for decrease of one standard error of the cohort-specific distribution of ln(antibody level) between the last and the first serological measurements, adjusted for all the variables in the table

CI, confidence interval; Ref, reference category; SD, standard deviation; HCW, healthcare worker
