# Supplementary figures and images for: Temporal trends of COVID-19 antibodies in vaccinated healthcare workers undergoing repeated serological sampling: An individual-level analysis within 13 months in the ORCHESTRA cohort
Source: Front Immunol. 2023 Jan 11;13:1079884. doi: 10.3389/fimmu.2022.1079884 (PMC9875291; doi:10.3389/fimmu.2022.1079884)

Supplementary Figure 1. Number of measurements by time since first vaccine dose and cohort


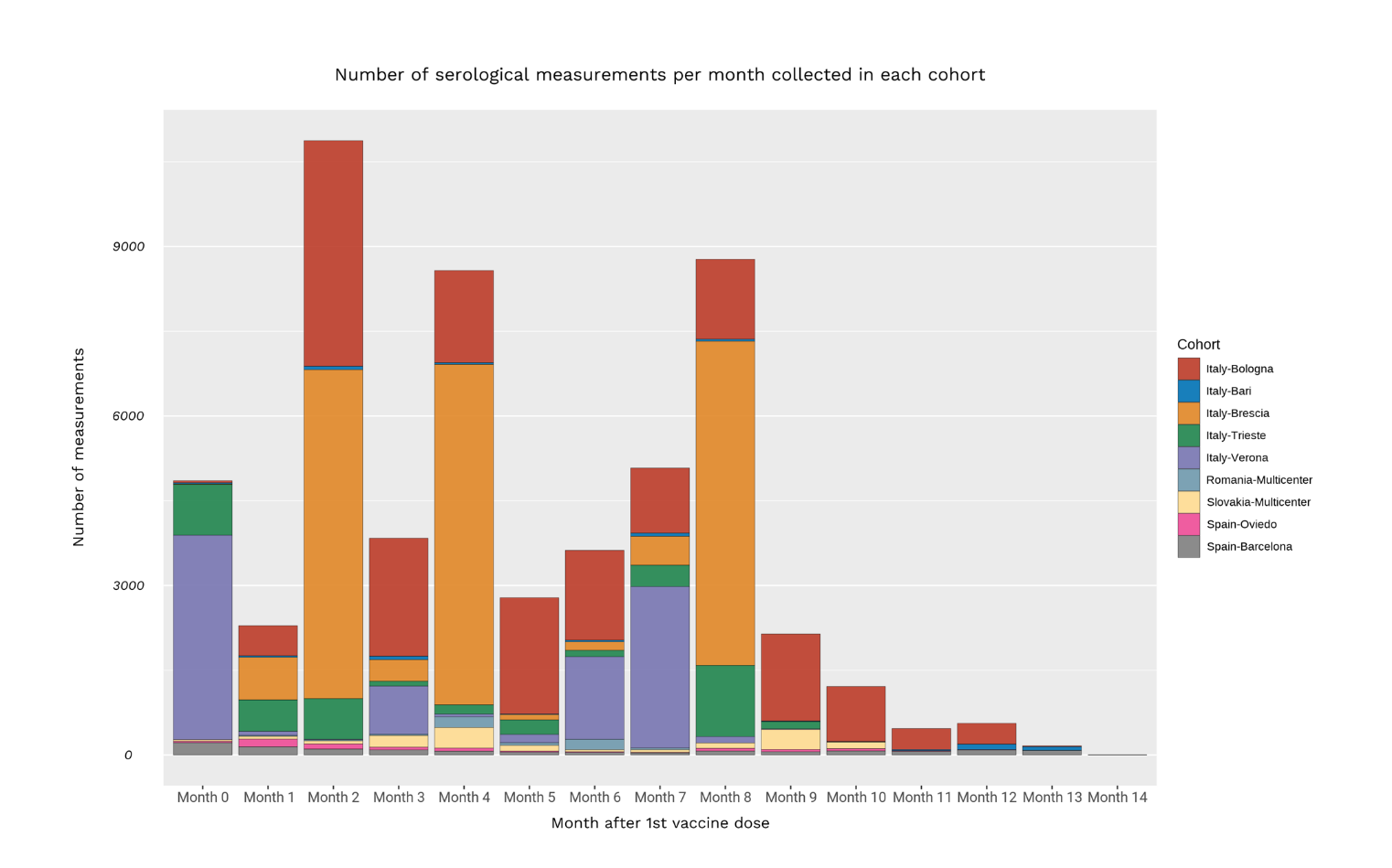

Supplement: Supplementary file 2 [file DataSheet_1.docx]
